# Transcriptional remodeling during metacyclogenesis in *Trypanosoma cruzi* I

**DOI:** 10.1080/21505594.2020.1797274

**Published:** 2020-07-27

**Authors:** Lissa Cruz-Saavedra, Gustavo A. Vallejo, Felipe Guhl, Louisa A. Messenger, Juan David Ramírez

**Affiliations:** a Grupo de Investigaciones Microbiológicas-UR (GIMUR), Departamento de Biología, Facultad de Ciencias Naturales, Universidad del Rosario, Bogotá, Colombia; b Laboratorio de Investigaciones en Parasitología Tropical, Facultad de Ciencias, Universidad del Tolima, Ibagué, Colombia; c Centro de Investigaciones en Microbiología y Parasitología Tropical (CIMPAT), Facultad de Ciencias, Universidad de Los Andes, Bogotá, Colombia; d London School of Hygiene and Tropical Medicine, London, UK

**Keywords:** *Trypanosoma cruzi*, metacyclic trypomastigotes, epimastigotes, transcriptomic, pathways

## Abstract

Metacyclogenesis is one of the most important processes in the life cycle of *Trypanosoma cruzi*. In this stage, noninfective epimastigotes become infective metacyclic trypomastigotes. However, the transcriptomic changes that occur during this transformation remain uncertain. Illumina RNA-sequencing of epimastigotes and metacyclic trypomastigotes belonging to *T. cruzi* DTU I was undertaken. Sequencing reads were aligned and mapped against the reference genome, differentially expressed genes between the two life cycle stages were identified, and metabolic pathways were reconstructed. Gene expression differed significantly between epimastigotes and metacyclic trypomastigotes. The cellular pathways that were mostly downregulated during metacyclogenesis involved glucose energy metabolism (glycolysis, pyruvate metabolism, the Krebs cycle, and oxidative phosphorylation), amino acid metabolism, and DNA replication. By contrast, the processes where an increase in gene expression was observed included those related to autophagy (particularly Atg7 and Atg8 transcripts), corroborating its importance during metacyclogenesis, endocytosis, by an increase in the expression of the AP-2 complex subunit alpha, protein processing in the endoplasmic reticulum and meiosis. Study findings indicate that in *T. cruzi* metacyclic trypomastigotes, metabolic processes are decreased, and expression of genes involved in specific cell cycle processes is increased to facilitate transformation to this infective stage.

## Introduction

*Trypanosoma cruzi* is a protozoan parasite that causes Chagas disease and is a serious public health problem in the Americas [1]. *T. cruzi* has a complex life cycle that alternates between triatomine insects and mammalian hosts, such as humans [2]. The parasite adopts four morphological forms that allows it to adapt to different microenvironmental stresses experienced during distinct life cycle stages [3].

The *T. cruzi* life cycle in mammals begins when metacyclic trypomastigotes (MTs), an infectious form, present in the vector’s feces reach peripheral blood through the skin wound caused by the triatomine during a blood meal and infect mononuclear cells, such as monocytes. In mononuclear cells, MTs differentiate into amastigotes, which are non-mobile replicative forms that undergo multiple rounds of division until finally transforming into cell-derived trypomastigotes (CDTs). The latter lyses the cells and migrate to infect other cells or tissues for which they have a high tropism [2,3]. The *T. cruzi* life cycle in insects begins when a triatomine bug ingests blood from mammals with CDTs circulating in the peripheral blood, which then differentiate into noninfective replicative epimastigotes (EPs). When the EPs reach the insect’s midgut, they continue migrating through the intestine, undergoing multiple rounds of replication until finally reaching the rectal ampulla, where they transform into MTs [2,3]. Metacyclogenesis is the process by which noninfective EPs transform into infectious MTs. Although some of the events carried out during metacyclogenesis remain unclear, the main stimulus is exposure of EPs to a poor nutritional environment that is rich in redox stress, leading to increased adenylate cyclase activity and consequent rise of intracellular cAMP levels in the parasite [4,5].

Metacyclogenesis is one of the most important and essential steps in the *T. cruzi* life cycle, in which a set of morphological, transcriptomic, proteomic, and metabolic changes allows the parasite to prepare for successful infection [6]. The most relevant morphological changes include modifying the position and shape of the nucleus and kinetoplast, which are associated with increased heterochromatin, followed by lengthening of the flagellum and elongation of the cytoplasm [7]. During this process, the proteome and phosphoproteome regulate proteins involved in transcription process, transialidases, mucin-associated surface proteins (MASPs), and dispersed gene family 1 (DGF-1) proteins, 12–24 hours after adhesion of EPs to the vector’s rectal cuticle [8]. Increased expression of mutagenic proteins has also been reported, including gp82s, calpain, and cruzipain, which are all involved in MT infectivity [9,10]. Regarding metabolic changes, increased proteolysis and metabolism, in response to redox stress, has been observed; these processes strongly influence metacyclogenesis and autophagy regulation [4,11–13].

Two previous studies have been carried out to understand genetic expression in EPs and MTs. The first of these performed by Minning *et al*., using microarrays, demonstrated an abundance of mRNA related to the morphological stage of *T. cruzi* during its life cycle, finding in MTs an upregulation of transcripts encoding transialidases and in EPs an increase in the expression of genes that participate in the histidine-to-glutamate pathway [14]. By comparison, analyses performed by Smircich *et al*., using SOLiD RNA-seq, confirmed the variation in expression profiles between EPs and MTs, characterized by an increase in the expression of genes mainly related to virulence (transialidases) [15]. To date, RNA-seq has been used to evaluate gene expression in EPs, amastigotes, and CDTs. Transcriptome remodeling occurs in these three morphological forms, with EPs characterized by highly expressed genes associated with energy metabolism, CDTs with evasion of the host immune system and membrane proteins, and amastigotes with genes involved in regulating the cell cycle [16]. These findings highlight the importance of modifying RNA expression profiles among different morphological forms of *T. cruzi*, enabling the parasite to adapt to the microenvironments it is exposed to during its life cycle [14,16]. In addition, transcriptomic analysis of EPs during growth curves has shown that during late-stage stationary phase, in response to nutritional stress, overall transcriptional expression decreases, while pre-adaptive upregulation of transialidases, nuclear-associated genes and those involved in flagellum processing occurs to enable transformation to the metacyclic form [17]. However, in this study, the EP and MT stages were not separated; thus, the authors could not determine which parasite stage contributed particular transcripts [17]. Other studies have focused on the relationship between transcriptomics of both the host cell and parasite, as well as virulence genes and remodeling during infection; however, these studies have thus far excluded the MT transcriptome [18–22].

Metacyclogenesis comprises a set of essential changes during the *T. cruzi* life cycle, which are crucial to facilitate the infection process and survival of the parasite outside the vector, as well as potentially contribute to differential virulence between strains. To date, there is still a paucity of information regarding the modifications the *T. cruzi* transcriptome undergoes during metacyclogenesis. Therefore, this study was conducted to evaluate the gene expression profiles of *Trypanosoma cruzi* I during metacyclogenesis *in vitro*.

## Results

### Metacyclogenesis curve

The MT concentration increased from day 1 post-culture in the three replicates evaluated, with an average concentration of 2.9 × 10^8^ trypomastigotes/mL. The highest MT concentration corresponded to an average of 5.35 × 10^8^ trypomastigotes/mL on day 7 post-culture; however, from day 7 onwards, the number of MTs decreased until reaching an average MT concentration of 1.75 × 10^8^ trypomastigotes/mL after 10 days post-infection (Figure S1). Analysis of data normality for the three biological replicates indicated a normal distribution. Thus, an analysis of variance (ANOVA) was performed, followed by multiple comparisons, which showed a difference between replicates. An ANOVA was performed to determine the first day of metacyclogenesis, which corresponded with day 4 post-culture (Figure S1).

### Gene expression profiles of epimastigotes and metacyclic trypomastigotes

**Figure 1.**
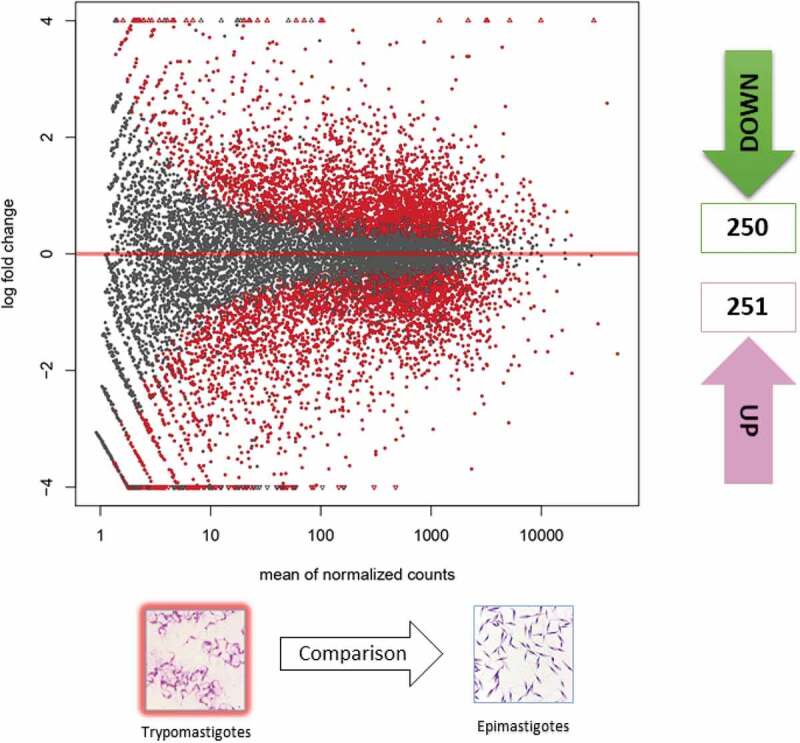
Gene expression of epimastigotes and metacyclic trypomastigotes. Volcano matrix to compare differentially expressed genes (DEGs) between metacyclic trypomastigotes (MTs) and epimastigotes (EPs), indicating the total number of down- and upregulated DEGs in metacyclic trypomastigotes.

RNA-sequencing of the eight transcriptomes included in this study generated an average of 56,997,358.81 and 21,980,185.92 sequencing reads (standard deviations of 6,350,528.951 and 1,377,905.656) for MTs and EPs, respectively. The results for each of the treatments and replicates are available in Table S1. The differentially expressed genes (DEGs) showed no statistically significant differences between the MT biological replicates (Figure S2B). However, we detected 18 DEGs corresponding to EPs, which differed among biological replicates. These genes were eliminated from the analysis to avoid bias when comparing the MT and EP profiles (Figure S2A). The DEGs differed significantly in MTs when compared with EPs; 250 DEGs were downregulated, and 251 were upregulated for the intersect between cufflinks and DESeq2 (Figure 1, Table S2). A heatmap showing the 50 genes that were the most down- and upregulated, and their respective logfold changes, is shown in Figure S3.

#### Gene ontology analysis

**Figure 2.**
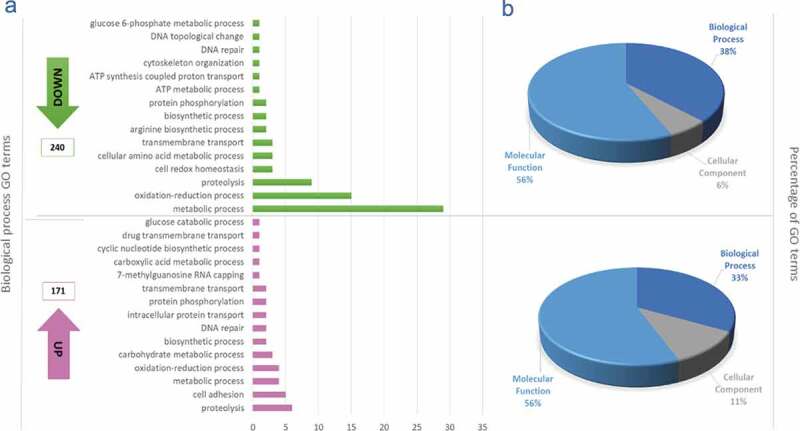
Gene ontology terms. (a) Gene ontology terms corresponding to the 15 most down and upregulated biological process from differential expressed genes obtained when were compared metacyclic trypomastigotes with epimastigotes; The ontological terms for the downregulated genes are in green and the ontological terms for the upregulated genes in pink. (b) Total proportions of ontology terms downregulated (upper) and upregulated (lower).

A total of 209 ontological terms were downregulated and 164 upregulated in MTs compared to EPs. The ontology that represented the highest number of down- and upregulated terms was related to molecular functions (65% and 58%, respectively), followed by biological processes, which had a greater proportion of downregulated genes (38%) compared to upregulated (33%), and finally, cellular components, with six terms downregulated and 11 upregulated (Figure 2, Table S3). Considering the importance of cellular processes throughout the *T. cruzi* life cycle and of course in metacyclogenesis and based on the objective of this study, we continued to describe in greater depth the cellular processes identified. The ontological term that grouped the largest number of downregulated genes corresponded to oxidation-reduction process (n = 15), followed by proteolysis (n = 9), and cell redox homeostasis, cellular amino acid metabolic process and transmembrane transport (with three genes each). By comparison, the proportion of upregulated genes with related ontological terms was lower, with proteolysis being the term that grouped the largest number of genes (n = 6), followed by cell adhesion (n = 5) and oxidation-reduction process (4) (Figure 2).

### Metabolic pathway reconstruction

From the GO terms, we selected the FASTA files and reconstructed the metabolic pathways, corresponding to the most down- and upregulated genes.

#### Glucose energy processes

**Figure 3.**
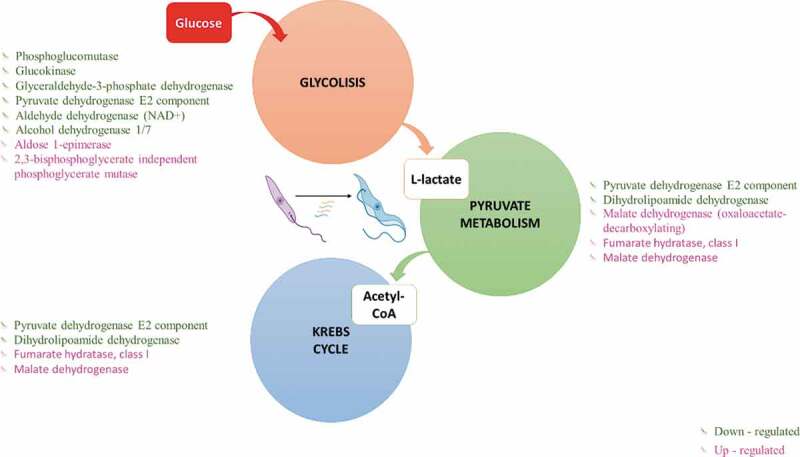
**Glucose energy processes**. Differentially expressed genes (DEGs) in Metacyclic trypomastigotes in comparison with epimastigotes in the metabolism of glycolysis, pyruvate, and the Krebs cycle (downregulated genes in green and upregulated genes in pink).

In MTs, six genes were downregulated for glycolysis, including the enzymes phosphoglucomutase (EC 5.4.2.2-TcSYL_0046700), glucokinase (EC 2.7.1.2), glyceraldehyde-3-phosphate dehydrogenase (EC 1.2.1.12-TcSYL_0011690), pyruvate dehydrogenase E2 component dihydrolipoamide acetyltransferase (EC 2.3.1.12-TcSYL_00734), aldehyde dehydrogenase (NAD+) (EC 1.2.1.3-TcSYL_0140060), and alcohol dehydrogenase 1/7 (EC 1.1.1.1). In addition, aldose 1-epimerase (EC 5.1.3.3-TcSYL_0172170) and 2,3-bisphosphoglycerate-independent phosphoglycerate mutase (EC 5.4.2.12-TcSYL_0108730) were upregulated (Figure 3).

Glucose degradation produces pyruvate via the glycolytic pathway. Three genes were upregulated in pyruvate metabolism, with malate dehydrogenase (oxaloacetate-decarboxylating) (EC 1.1.1.38-TcSYL_0047970) exclusively related to pyruvate metabolism (Figure 3). Pyruvate metabolism allows acetyl-CoA formation, which is necessary for Krebs cycle progression; two genes involved in pyruvate metabolism/Krebs cycle were downregulated, pyruvate dehydrogenase E2 component (dihydrolipoamide acetyltransferase) (EC:2.3.1.12-TcSYL_0073490) and dihydrolipoamide dehydrogenase (EC:1.8.1.4-TcSYL_0111890), and two genes involved in the pyruvate metabolism/Krebs cycle were upregulated, fumarate hydratase, class I (EC 4.2.1.2-T40S), and malate dehydrogenase (EC 1.1.1.37) (Figure 3)

#### Amino acid metabolism

Next, we evaluated the differential metabolism of amino acids in MTs.

For alanine, aspartate, and glutamate metabolism, three genes were downregulated and one was upregulated. The downregulated genes corresponded to alanine transaminase (EC 2.6.1.2-TcSYL_0178360), glutamate dehydrogenase (NADP+) (EC 1.4.1.4-TcSYL_0074000) and 1-pyrroline-5-carboxylate dehydrogenase (EC 1.2.1.88-TcSYL_0001430); aspartate aminotransferase, cytoplasmic (EC: 2.6.1.1-TcSYL_0131200) was upregulated.

For glycine, serine and threonine metabolism, three genes were downregulated: glycine hydroxymethyltransferase (EC: 2.1.2.1 – TcSYL_0027590), dihydrolipoamide dehydrogenase (EC: 1.8.1.4-TcSYL_0111890) and glycine C-acetyltransferase (EC: 2.3. 1.29-TcSYL_0103590); and one upregulated: 2,3-bisphosphoglycerate-independent phosphoglycerate mutase (EC: 5.4.2.12- TcSYL_0108730). For tyrosine metabolism, tyrosine aminotransferase (EC:2.6.1.5-TcSYL_0012630), alcohol dehydrogenase 1/7 (EC:1.1.1.1), and 4-hydroxy-2-oxoheptanedioate aldolase (EC:4.1.2.52- TcSYL_0019630) were downregulated and aspartate aminotransferase, cytoplasmic (EC:2.6.1.1- TcSYL_0131200) was upregulated.

Regarding cysteine and methionine metabolism, three genes were downregulated; 5ʹ-methylthioadenosine phosphorylase (EC 2.4.2.28-TcSYL_0163070) is expressed only in the metabolism of cysteine and methionine, and S-adenosylmethionine synthetase (EC 2.5.1.6-TcSYL_0140680), and tyrosine aminotransferase (2.6.1.5-TcSYL_0012630) are also related to other metabolic pathways. In addition, three genes were upregulated: aspartate aminotransferase, cytoplasmic (EC 2.6.1.1-TcSYL_0131200), thiosulfate/3-mercaptopyruvate sulfurtransferase (EC:2.8.1.1 2.8.1.2-TcSYL_0079450) and malate dehydrogenase (K00026).

Finally, down- and upregulated genes related to valine, leucine, and isoleucine degradation were increased. Two downregulated genes were dihydrolipoamide dehydrogenase (EC 1.8.1.4-TcSYL_0111890) and 2-oxoisovalerate dehydrogenase E2 component (dihydrolipoyl transacylase) (EC 2.3.1.168-TcSYL_0062200); only one gene expressed in this pathway was upregulated: 3-methylcrotonyl-CoA carboxylase alpha subunit (EC 6.4.1.4-TcSYL_0057610).

#### Cellular processes

**Figure 4.**
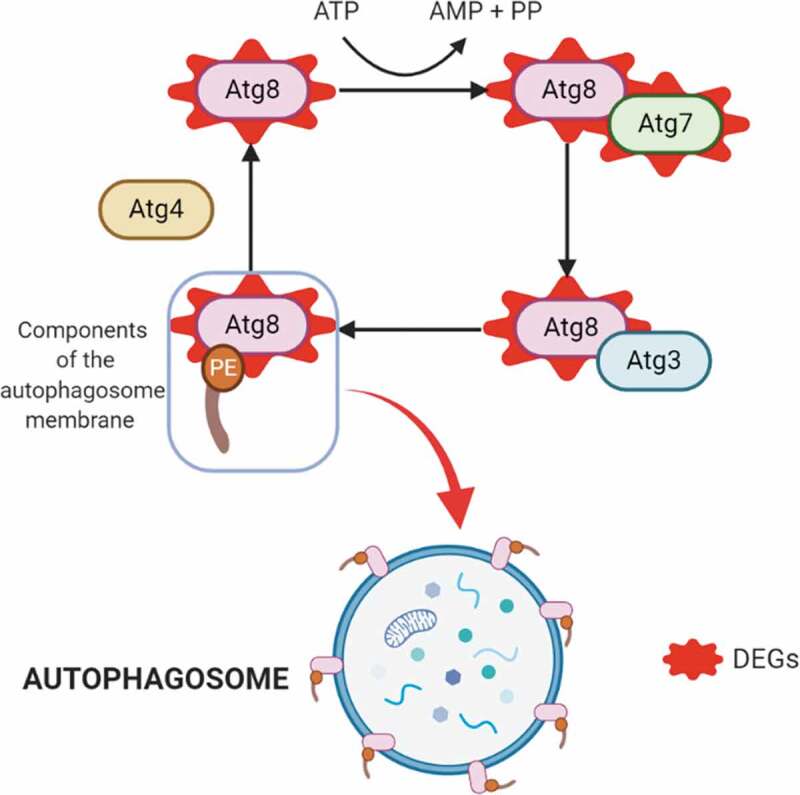
Autophagy process. Upregulated DEGs in autophagy (in red). The cysteine protease Atg4 cuts the arginine residue in the C-terminal part of Atg8, and immediately Atg8 is transferred to Atg7 and Atg3, and finally to the substrate phosphatidylethanolamine (PE); this complex (Atg8-PE) is part of the autophagosome membrane components.

Consistent with the morphological modifications that *T. cruzi* undergoes during metacyclogenesis, three genes related to regulation of the actin cytoskeleton were upregulated: phosphatidylinositol-4,5-bisphosphate 3-kinase catalytic subunit alpha/beta/delta (EC: 2.7.1.153 – TcSYL_0025520), actin-related protein 2/3 complex, subunit 5, and serine/threonine-protein phosphatase PP1 catalytic subunit (EC: 3.1.3.16-TcSYL_0044050). Endocytosis-related genes were also increased in expression, with two genes upregulated from this process: AP-2 complex subunit alpha (TcSYL_0201950) and ARP 2/3 actin-related protein 2/3 complex, subunit 1A/1. Likewise, protein processing in the endoplasmic reticulum was upregulated (protein transport protein SEC61 subunit alpha, calreticulin (TcSYL_0030690), and DnaJ homolog subfamily A member 1). Previous studies have demonstrated the importance of autophagy activation as a stimulus for metacyclogenesis, and the presence of the autophagy protein TcAtg8 that triggers metacyclogenesis under conditions of nutritional depression [13]. In this study, two genes in this process were upregulated: ubiquitin-like modifier-activating enzyme Atg7 (TcSYL_0008790) and GABA (A) receptor-associated protein Atg8 (TcSYL_0079440) (Figure 4).

#### DNA processes

Two RNA-encoding proteins involved in DNA replication were downregulated, corresponding to proliferating cell nuclear antigen (PCNA-TcSYL_0064140) and replication C subunit 3/5 (TcSYL_0116350); cell cycle cyclin-dependent kinase regulatory subunit CKS1 (TcSYL_0112190) and MDR1 cohesin complex subunit SCC1 (TcSYL_0162370) were also downregulated. Two genes were upregulated which are involved in meiosis, adenylate cyclase (Cyr1) (TcSYL_0107750) and serine/threonine-protein phosphatase PP1 catalytic subunit (Glc7); however, these genes are not specific to this process and participate in other metabolic pathways (TcSYL_0044050).

## Discussion

*T. cruzi* presents a peculiar transcriptional control, where polycistron mRNAs that include genes that are not necessarily related, are transcribed by RNA polymerase II. mRNA maturation involves a process of trans-splicing, with gene expression being regulated at a posttranscriptional level [23]. This complex process suggests the need to unveil the transcriptomic features of this parasite in terms of understanding its biology per se.

One of the most important processes in the life cycle of *T. cruzi* is metacyclogenesis. This process allows the parasite to acquire all necessary metabolic and structural features to differentiate from a noninfective (EPs) to an infective stage (MTs) and overcome the harsh environmental changes the parasite faces throughout its life cycle [6]. Previous studies have evaluated how starvation, redox stress, proteome modifications, the phosphoproteome, and expression of proteins involved in virulence, are the main stimuli in inducing metacyclogenesis [4–6,10,12]. This study was conducted to evaluate transcriptome remodeling in MTs with respect to EPs using RNA-seq. The MT transcriptome differed significantly from the EP transcriptome, results that have previously been observed using microarrays and SOLiD RNA-seq [14,15]. This was expected because of the morphological and biochemical differences exhibited by MTs, including the modified shape of the nucleus and kinetoplast, changes in chromatin compaction, repositioning of the flagellum–kinetoplast complex, decreases in reservosome size and an increase in specific proteins related to virulence and infective capacity [7,22].

We found that one of the most affected metabolic pathways in MTs was energy metabolism from glucose, in which we identified downregulated genes in glycolysis, pyruvate metabolism, the Krebs cycle, and oxidative phosphorylation. These genes are likely downregulated because of the different nutritional requirements of MTs. This observation was verified by comparing EP transcriptomes in logarithmic phase and EPs in stationary phase, which indicated that regulation of these processes influences the parasite’s adaptation to microenvironments under nutritional stress (Figure 3) [4,11]. In *T. cruzi*, glycosomes are organelles that have the enzymatic content of different metabolic pathways, thus allowing compartmentalization of different processes [24]. Previous studies in *T. brucei* have shown a decrease in the glycolytic pathway glycosomes in the infective form of the parasite in the vector, allowing the parasite a rapid regulation of the metabolism in response to low glucose environments [13]. An increase in the number of down-regulated genes in MTs, the infective form of *T. cruzi* present in the vector, is consistent with these findings; however, the presence of genes coding for fumarate hydratase, class I and malate dehydrogenase up-regulated in MTs, both enzymes being involved in the glycolytic branch and active as a consequence of a decrease in pyruvate synthesis, which would possibly explain the down-regulation of pyruvate hydrogenase observed in MTs [25]. The activation of the glycolytic arm and its relationship with the decrease in pyruvate has already been previously documented in *T. cruzi* CDTs in response to the presence of a cell matrix, the results observed here could infer the presence of these regulatory processes in other stages of *T. cruzi* but future mechanistic studies must be conducted to fulfill this hypothesis [26].

Amino acids play important roles in the parasite lifecycle, including in processes related to survival, death, differentiation, and evasion of the host immune system [27]. One characteristic of the MT transcriptome was decreased amino acid metabolism, which may be a consequence of starvation faced by the parasite. In addition, the activation of amino acid catabolism as an energy and carbon source has been demonstrated in epimastigotes in the stationary phase, allowing this metabolic plasticity to adapt to the nutritional conditions it faces [11]. Triatomine artificial urine medium is used in some studies to initiate metacyclogenesis *in vitro* and is supplemented with amino acids such as proline, glutamate, and aspartate, equally present at the distal portion of the triatomine’s [28]. Previous studies have shown the importance of L-proline in energy metabolism during metacyclogenesis in addition to its role during oxidative stress and against other different abiotic and biotic stresses [29]. This may indicate that during this phase of the *T. cruzi* life cycle, although these amino acids are not synthesized, the parasite can use previously synthesized reserves. Future Analysis of the amino acid composition during metacyclogenesis and in mature MTs could shed light on amino acid regulation during these stages of the *T. cruzi* life cycle [30]. In contrast, upregulation of the genes involved in synthesizing methionine and cysteine showed a variable expression. Studies of trypanosomatids, such as *Trypanosoma brucei brucei* and *Crithidia fasciculata*, have shown that these parasites can recycle methionine from methylthioadenosine, a product derived from the polyamine synthetic pathway found in EP reservosomes in *T. cruzi*, which may explain its availability in MTs [31,32].

Autophagy allows *T. cruzi* to withstand nutritional stress during metacyclogenesis and amastigogenesis [13,33,34]. The autophagy process involves (Atg)-related proteins and begins when the cysteine protease Atg4 cuts the arginine residue in the C-terminal part of Atg8, and immediately, the protein Atg8 is transferred to Atg7 and later to Atg3, to finally be transferred to the substrate phosphatidylethanolamine (PE). This complex (Atg8-PE) is a principal part of the autophagosome membrane components, making Atg8 one of the best autophagy markers [13]. We found two upregulated genes involved in autophagy, including Atg7 (Figure 4). One upregulated gene was Atg7. Although this gene has not been previously studied in *T. cruzi*, it functions by interrupting autophagy in the bloodstream stages of *Trypanosoma brucei*; thus, it is possible that Atg7 fulfills a similar function in *T. cruzi* MTs. The presence of both Atg7 and Atg8 confirms the importance of autophagy during metacyclogenesis, future studies should conduct longer sequential RNA-seq analyses to further investigate how autophagy is exploited by MTs and characterize the autophagy-related genes that are expressed (Figure 4) [13,35].

Endocytosis in *T. cruzi* is restricted to two specialized invaginations around the base of the flagellum; these are the flagellar pocket membrane and the cytostome, the latter being present only in the replicative forms of the parasite (EPs and AMs) [36]. In this study, upregulated genes included AP-2 complex subunit alpha (AP-2), a clathrin-associated protein. Within this gene family, AP-1 decreases the proliferation and differentiation of EP into MTs, while AP-3 is essential for *T. brucei* growth and virulence [37]. Previous studies evaluated AP-2 expression in EPs and CDTs of *T. cruzi*; thus, we verified the ID TcSYL_0201950 that codes for this protein in our dataset (Table S2). We observed that although EPs express this protein, its expression is greater in MTs [38,39]. To what extent the increased expression of this protein can confer certain properties to MTs remains unknown, but these results suggest that MT expression levels are regulated to modify an entire metabolic pathway. Endocytosis, in addition to being an important process in regulating the parasite’s nutritional requirements, may play a role in regulating the transport of proteins involved in other MT functions such as virulence; these proteins may be previously synthesized and carried to the membrane by endocytosis and vacuole-linked transport.

A recent genomics study of *T. cruzi* reported meiotic sex as a mechanism of genetic exchange; however, the ability for recombination in *T. cruzi* has been heavily debated over the past few decades, and the population of this parasite has been mainly characterized as clonal [40–44]. MT transcriptome analysis demonstrated the presence of two upregulated genes associated with meiosis, and bioinformatics studies of the sequenced genomes have demonstrated that 40–60% of the human/yeast signaling pathway involved in meiosis in trypanosomatids, such as *Leishmania* and *Trypanosoma*, is conserved [42]. This is the first study where the expression of genes related to meiosis in some stage of *T. cruzi* has been demonstrated, despite this, the genes found may participate in other cellular processes, which is why there is no absolute certainty of activating the meiotic process during metacyclogenesis [45]. Despite this, the absence of other specific meiotic proteins could be related to the time at which the RNA was extracted from the MTs (4 days); daily monitoring of gene expression for additional days could provide more information about this cellular process. Intriguingly, the first report of genetic exchange in *T. cruzi* showed that this biological process occurs in CDTs in Vero cells, suggesting the presence of meiosis-related proteins during some parts of the *T. cruzi* life cycle [46]. One of the upregulated genes was the adenylate cyclase (Cyr1), which induces the production of cAMP in response to redox stress; *in vitro* analyses have shown an increase in the levels of this enzyme during metacyclogenesis. Furthermore, the receptor-type adenylate cyclase putative gene has been reported to be upregulated in mature MTs, which together suggests the importance of this enzyme in metacyclogenesis [4,14]. Future studies should examine the transcriptome of the entire *T. cruzi* life cycle to determine which stage has the capacity for recombination and to determine the true recombination mechanism(s) used by *T. cruzi*. This particular analysis was conducted on TcI (*T. cruzi* I) strains, and other DTUs (Discrete typing units) and their capacity for recombination should also be considered.

## Materials and methods

### Parasite culture and metacyclogenesis curve

EPs of the *T. cruzi* strain MHOM/CO/04/MG (*T. cruzi* I) were cultured in liver infusion tryptose medium (LIT) supplemented with 10% fetal bovine serum at 26°C. We followed a previously described protocol to construct metacyclogenesis curves [47]. In summary, three biological replicates were used, and 1 × 10^8^ EP/mL in the logarithmic phase were cultured in LIT medium supplemented with 10% fetal bovine serum. The parasites were quantified by counting in a Neubauer chamber daily for 10 days, and this process was repeated three times. Slides of the parasites were stained with 10% Giemsa and observed under optical microscopy to visually identify EP and MT forms. Three hundred parasites were classified as EP or MT based on the locations of the nucleus and kinetoplast and on the elongated flagellum in MTs. Three hundred cells were counted per sample. The results were tabulated in Excel over 10 days.

### Determining the first day of metacyclogenesis and statistical analysis

To determine the beginning of metacyclogenesis, we obtained the daily MT concentration from the total parasite concentration and the percentage of trypomastigotes observed on the Giemsa-stained slides. We used three replicates to determine the concentration of trypomastigotes in the sample. A Shapiro–Wilk test was performed to observe normality for the MT data. A Kruskal–Wallis test followed by Dunn’s multiple comparison test were used to determine the first day of metacyclogenesis, which corresponded to the day on which the MT number was significantly increased. Graphs were constructed based on the number of MTs and EPs per day. Statistical analyses were performed and graphs were constructed using GraphPad Prism, version 7.4 (GraphPad Software Inc., La Jolla, CA, USA).

### Purification of MTs and RNA extraction

EP/MT cultures in LIT medium were selected on the previously calculated first day of metacyclogenesis to purify the MTs; the parasites were washed twice with 1X phosphate-buffered saline (PBS); then, resin-exchange sepharose-DEAE chromatography was performed following the protocol described by Cruz-Saavedra *et al*. 2017 [47]. The eluate containing trypomastigotes was obtained, then washed again with 1X PBS, and RNA was extracted using the RNeasy Plus Mini Kit (Qiagen, Düsseldorf, Germany) following the manufacturer’s protocols. RNA quality and quantity was evaluated using three parameters: integrity by 2% agarose gel electrophoresis, 1 mg/mL concentration using NanoDrop™ 2000/2000 c spectrophotometers (Thermo Fisher Scientific Inc., CA, USA) and purity using indexes 260/280 and 230/260, which each were ~2.0. To compare gene expression profiles from the MTs against the EPs, we extracted RNA from the logarithmic phase for EPs from a culture in LIT, which was washed twice with PBS; then, the aforementioned RNA extraction protocol was followed.

### Preparation of the libraries and RNA sequencing

Extracted RNA was sent to Novogene Bioinformatics Technology Co., Ltd., Beijing, China, to construct the libraries and for sequencing using the Illumina HiSeq X-TEN platform. As an initial step, prior to library preparation, the commercial company verified the RNA quality using an agarose gel and Qubit analysis; only samples that passed these quality controls were included in the sequencing run. To construct the libraries, the extracted RNA was enriched using oligo-beads (dT), and rRNA was removed using a Ribo-Zero kit (Illumina, California, United states). Finally, RNA was randomly fragmented using a fragmentation buffer. The cDNA was produced using a strand-specific TrueSeq RNA-seq Library Prep kit (Illumina, California, United states), following the supplier’s instructions. RNA libraries were prepared with an insert size of 350 bp. The size of each read was 2 × 150 bp. Read quality was analyzed using FastQC software (https://www.bioinformatics.babraham.ac.uk/projects/fastqc), which considered 10 parameters, including per base sequence quality, per sequence GC content, and Kmer content. In order to improve the quality of the reads, Novogene performed filtering of low-quality reads to remove those that had adapters, reads containing N > 10% (N represents a base that cannot be determined), and those with low-quality scores (Qscore ≤5). Finally, FastQC analysis was performed again using the previously mentioned parameters based on the filtered reads. Two biological replicates and two technical replicates were included per experiment.

### Mapping and differential expression analysis

Eight paired-end samples, corresponding to two biological replicates and two technical replicates per stage were individually aligned using TopHat – v2.1.0 version, which uses bowtie as an alignment engine but has the ability to recognize splicing sites. TopHat was run using default parameters, and the FASTA file of the reference genome of *Trypanosoma cruzi* Sylvio X10-1 version 43, available in EupathDB, was obtained to assemble the samples [48,49]. Differential expression analysis was performed using two methods: cufflinks and HTseq/DESeq2. The bam files obtained from the TopHat assembly were used for mapping in Cufflinks, the gff annotation file of the *T. cruzi* reference genome, Sylvio X10-1 – 43 version, also available in EupathDB, was used to obtain the location of genes in the reference genome. Next, the cuffmerge tool under the “g” and “s” options was used to join files, including those to compare the replicates for each stage (EPs and MTs) and between stages. Finally, differentially expressed genes (DEGs) were identified using the cuffdiff tool, including the parameters “-p”, “L”, and “u.” Fragments per kilobase of exon per million fragments mapped (FPKM) was used to normalize the expression, the *p*-value was calculated from the log2 fold change obtained, and finally, the value was obtained from the correction of the *p*-value. Genes that presented a *q*-value <0.05 were considered statistically significant [3,49]. Regarding the second methodology used, the Python Toolbox HTseq-count tool was used to transform genetic depth information into a count of readings by gene overlapping using the gff annotation file of the *T. cruzi* reference genome, Sylvio X10-1-43 version; .txt output files were obtained for each replicate for each stage (EPs and MTs) [50]. The DESeq Bioconductor package version 1.26.0 was used to determine DEGs, data normalization was performed using the median of ratios method, and the default parameters were followed; transcripts with a log2 fold change >2 and that presented a statistically significant differential expression (*p*adj = <0.05) were selected [51]. DEGs identified by Cufflinks and DESeq2 were classified as downregulated or upregulated. Outputs of both methodologies were compared and selected gene IDs, identified with both tools, were submitted to the virtual tool Venny 2.1, and taken forward for analysis [52].

### Gene ontology and pathway reconstruction

The gene IDs for down- and upregulated DEGs, obtained from both Cufflink and DESeq2, were submitted to the EupathDB TriTrip online tool in two different lists to obtain the gene ontology (GO) terms for enrichment analysis [53,54]. To reconstruct protein pathways, a FASTA output file was obtained from the IDs of the down- and upregulated DEGs submitted to EupathDB with protein-coding sequences. Each gene FASTA file was subjected to the KEGG Automatic Annotation Server (KAAS) tool, selecting the homology search algorithm GHOSTX and the GENES dataset for kinetoplasts available in the manually curated KEGG GENES database [55,56]. The html file obtained was analyzed under the “Pathway” map option to search the pathways that presented a greater KEGG orthology from the DEGs. This tool allowed maps for each differentially regulated pathway to be predicted [55]. KAAS annotates genes by comparing them against the cured KEGG bases using algorithms such as BLAST or GHOSTX; the results are grouped according to the metabolic pathways in which they are activated allowing them to be visualized graphically. The Biorender tool was used (https://biorender.com/) to reconstruct the autophagy metabolic pathway.

## Conclusions

In conclusion, transcriptomic gene expression differed significantly between *T. cruzi* EPs and MTs. In MTs processes related to ATP production from glucose were downregulated, including pathways for glycolysis, pyruvate metabolism, and the Krebs cycle, likely in response to increased nutritional stress. Changes to the microenvironmental composition may act as a stimulus for increased endocytosis in *T. cruzi* in response to nutrient deprivation, which in turn promotes endocytosis to transport virulence-related proteins in MTs, such as Gp82 and Gp90. In addition, during metacyclogenesis, energy deficiency leads to a decrease in amino acid metabolism, which may be compensated by increased availability of reservosomes, to provide these amino acids and essential proteins for MTs. Autophagy-related genes are also upregulated, while those involved in DNA replication are downregulated, to facilitate transformation to a nonreplicative form. Future studies should also consider parasitic genetic diversity (DTUs); improved understanding of DNA editing throughout the *T. cruzi* lifecycle will be pivotal for the development of novel drugs and potential vaccines to reduce the current burden of Chagas disease.

## Supplementary Material

Supplemental Material

## Data Availability

All data employed in this paper are available in the European Nucleotide Archive (ENA) under PRJEB33521 study accession (https://www.ebi.ac.uk/ena/data/view/PRJEB33521).
