# Supplementary material for: Transcriptional remodeling during metacyclogenesis in Trypanosoma cruzi I
Source: Virulence. 2020 Jul 27;11(1):969–80. doi: 10.1080/21505594.2020.1797274 (PMC7549971; doi:10.1080/21505594.2020.1797274)
Supplement: Supplemental Material [file KVIR_A_1797274_SM7171.zip › Figure S3.pdf]

Down-regulated		Up-regulated	
TcSYL_0042320	-9,94644E+14	9,95892E+14	TcSYL_0094590
TcSYL_0109330	-9,92865E+14	9,91006E+14	TcSYL_0092090
TcSYL_0042510	-9,86236E+14	9,85426E+14	TcSYL_0203380
TcSYL_0043520	-9,82935E+14	9,66402E+14	TcSYL_0146450
TcSYL_0121250	-9,73670E+14	9,62026E+14	TcSYL_0112760
TcSYL_0042800	-9,72591E+14	9,54554E+14	TcSYL_0174470
TcSYL_0074680	-9,72556E+14	9,45925E+14	TcSYL_0045340
TcSYL_0096870	-9,70131E+14	9,42134E+14	TcSYL_0073440
TcSYL_0142680	-9,63428E+14	9,28700E+14	TcSYL_0001090
TcSYL_0181830	-9,58092E+14	9,26693E+14	TcSYL_0200790
TcSYL_0084870	-9,57777E+14	9,19340E+14	TcSYL_0123030
TcSYL_0048090	-9,55732E+14	9,04323E+14	TcSYL_0188280
TcSYL_0078820	-9,49666E+14	8,96298E+14	TcSYL_0115400
TcSYL_0096350	-9,43347E+14	8,92749E+14	TcSYL_0179700
TcSYL_0047110	-9,42161E+14	8,62460E+14	TcSYL_0138270
TcSYL_0138590	-9,38979E+14	8,54716E+14	TcSYL_0094580
TcSYL_0062190	-9,38115E+14	8,52846E+14	TcSYL_0007990
TcSYL_0142770	-9,31508E+14	8,49186E+14	TcSYL_0113510
TcSYL_0195680	-9,22916E+14	8,32890E+14	TcSYL_0030690
TcSYL_0091930	-9,16103E+14	8,31006E+14	TcSYL_0001810
TcSYL_0063430	-9,13302E+14	8,29151E+14	TcSYL_0200910
TcSYL_0014470	-9,11802E+14	8,27078E+14	TcSYL_0027680
TcSYL_0116350	-9,09971E+14	8,24518E+14	TcSYL_0108650
TcSYL_0048290	-9,06370E+14	8,05658E+14	TcSYL_0094600
TcSYL_0146090	-9,05897E+14	8,05051E+14	TcSYL_0109340
TcSYL_0106950	-9,03140E+14	7,98025E+14	TcSYL_0146350
TcSYL_0048370	-9,00185E+14	7,92659E+14	TcSYL_0057610
TcSYL_0005490	-8,88267E+14	7,81079E+14	TcSYL_0097480
TcSYL_0086570	-8,84482E+14	7,76873E+14	TcSYL_0113440
TcSYL_0043130	-8,80696E+14	7,24414E+14	TcSYL_0157160
TcSYL_0122190	-8,77361E+14	7,12598E+14	TcSYL_0114960
TcSYL_0117700	-8,75615E+14	6,95958E+14	TcSYL_0050050
TcSYL_0118140	-8,71284E+14	6,69781E+14	TcSYL_0001380
TcSYL_0043650	-8,70562E+14	6,44291E+14	TcSYL_0122980
TcSYL_0001160	-8,63796E+14	6,40147E+14	TcSYL_0086690
TcSYL_0030730	-8,62545E+14	6,33571E+14	TcSYL_0179130
TcSYL_0193870	-8,56917E+14	6,12196E+14	TcSYL_0159980
TcSYL_0075560	-8,54027E+14	5,36078E+14	TcSYL_0044050
TcSYL_0191570	-8,39750E+14	4,79471E+14	TcSYL_0200640
TcSYL_0171190	-8,26721E+14	4,11813E+14	TcSYL_0060670
TcSYL_0063520	-8,23054E+14	3,63639E+14	TcSYL_0140840
TcSYL_0004670	-8,21757E+14	3,61924E+14	TcSYL_0008880
TcSYL_0005950	-8,18172E+14	3,59952E+14	TcSYL_0109690
TcSYL_0201790	-8,11937E+14	3,59023E+14	TcSYL_0027040
TcSYL_0106900	-8,04231E+14	3,50652E+14	TcSYL_0204540
TcSYL_0112920	-8,03341E+14	3,39179E+14	TcSYL_0004850
TcSYL_0111840	-7,93271E+14	3,37698E+14	TcSYL_0158970
TcSYL_0146630	-7,90574E+14	3,26248E+14	TcSYL_0084130
TcSYL_0085060	-7,87911E+14	3,1314E+14	TcSYL_0074330
