# Supplementary material for: Transcriptional remodeling during metacyclogenesis in Trypanosoma cruzi I
Source: Virulence. 2020 Jul 27;11(1):969–80. doi: 10.1080/21505594.2020.1797274 (PMC7549971; doi:10.1080/21505594.2020.1797274)
Supplement: Supplemental Material [file KVIR_A_1797274_SM7171.zip › Supplementary Materials.docx]

**Supplementary Materials:**

Figure S1: Metacyclogenesis curve. Metacyclogenesis curve during 10 days in liver infusion culture (LIT) and the first day of metacyclogenesis. (pdf);

Figure S2: Volcano matrix of DEGs in the biological replicates. Comparison of biological replicates for metacyclic trypomastigotes (MTs) and epimastigotes (EPs). (pdf);

Figure S3. Heatmap of the 50 most down- and upregulated genes, in the center of the bars you can see the value of the logfold changes for each gene.

Table S1: Read per transcriptome.;

Table S2: DEG statistics. Downregulated and upregulated DEGs for metacyclic trypomastigotes (MTs) against epimastigotes (EPs) using the cuffdiff tool in Cufflinks.(xlsx);

Table S3: Gene ontology (GO). GO terms for the downregulated and upregulated genes for metacyclic trypomastigotes (MTs). (xlsx)
