# Supplementary figures and images for: Transcriptional remodeling during metacyclogenesis in Trypanosoma cruzi I
Source: Virulence. 2020 Jul 27;11(1):969–80. doi: 10.1080/21505594.2020.1797274 (PMC7549971; doi:10.1080/21505594.2020.1797274)

# Metacyclogenesis curve

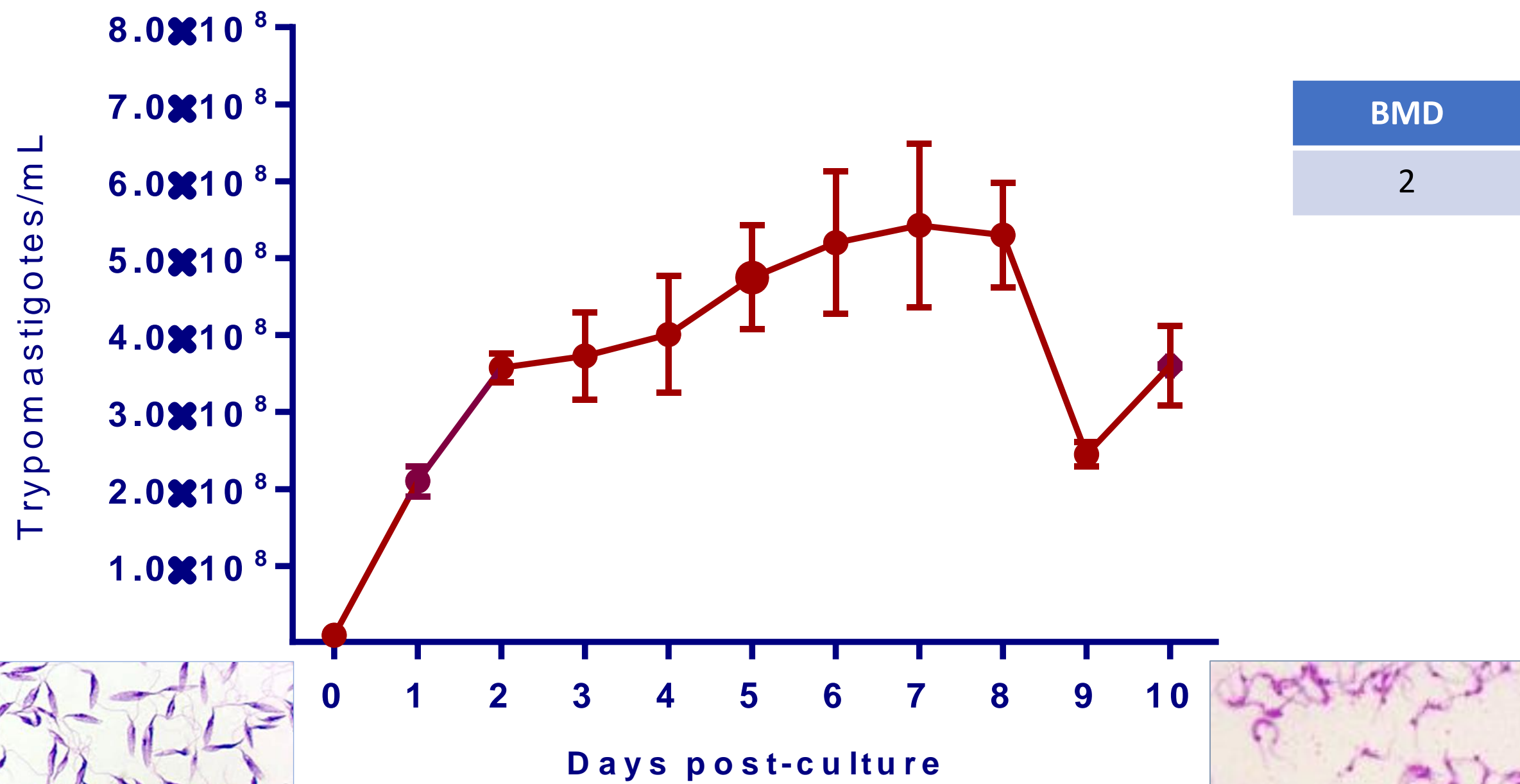

**BMD:** the begging of metacyclogenesis day

Supplement: Supplemental Material [file KVIR_A_1797274_SM7171.zip › Figure S1.pdf]

A

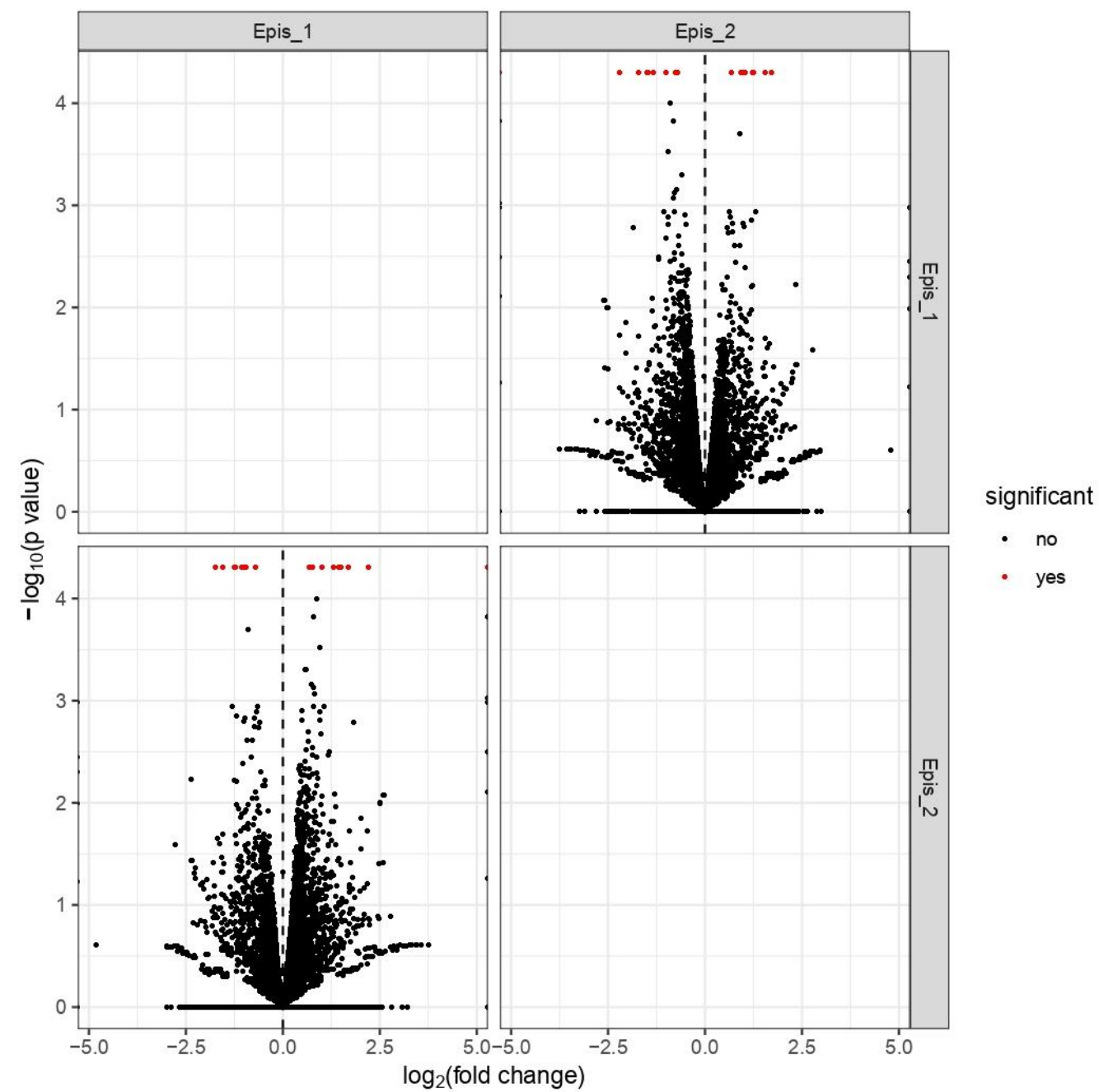

B

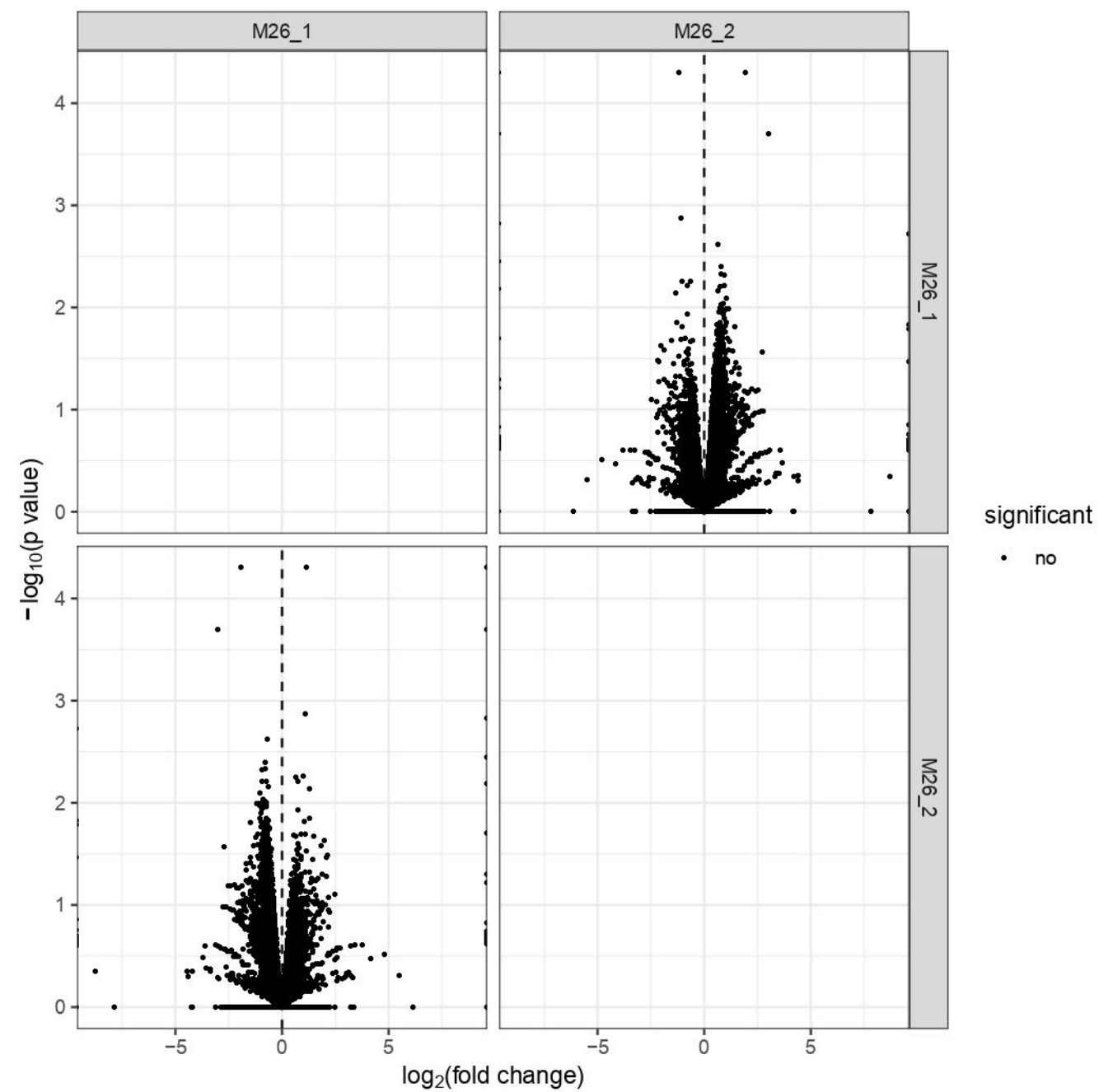

Supplement: Supplemental Material [file KVIR_A_1797274_SM7171.zip › Figure S2.pdf]
